# Crystal structure of (*Z*)-2-(1-benzyl-2-oxoindolin-3-yl­idene)-*N*-phenyl­hydra­zine-1-carbo­thio­amide

**DOI:** 10.1107/S2056989015002248

**Published:** 2015-02-07

**Authors:** G. Vimala, J. Haribabu, R. Karvembu, B. V. N. Phani Kumar, A. SubbiahPandi

**Affiliations:** a Department of Physics, Presidency College (Autonomous), Chennai 600 005, India; b Department of Chemistry, National Institute of Technology, Trichy 620 015, India; c Chemical Physics Laboratory, Central Leather Research Institute, Adyar, Chennai 600 020, India

**Keywords:** crystal structure, thio­semicarbazones, hydrazine, carbo­thio­amide, 2-oxoindolin-3-yl­idene, hydrogen bonding, C—H⋯π inter­actions

## Abstract

The title compound, C_22_H_18_N_4_OS, crystallized with four independent mol­ecules (*A*, *B*, *C* and *D*) in the asymmetric unit. All four mol­ecules have a *Z* conformation about the C=N bond with the benzyl ring being inclined to the indoline ring mean planes by 73.4 (2), 77.9 (2), 73.2 (2) and 77.2 (2)° in mol­ecules *A*, *B*, *C* and *D*, respectively. In mol­ecules *A* and *B*, the phenyl ring is inclined to the mean plane of the indoline ring mean plane by 12.0 (2) and 12.2 (2)°, respectively. However, in mol­ecules *C* and *D*, the same dihedral angles are larger, *viz.* 37.3 (2) and 36.4 (2)°, respectively. Consequently, the benzyl and phenyl rings are almost normal to one another in mol­ecules *A* and *B* [dihedral angles = 80.3 (3) and 87.1 (3)°, respectively], while in mol­ecules *C* and *D*, the same dihedral angles are only 48.8 (2) and 43.8 (3)°, respectively. There is an intra­molecular N—H⋯O hydrogen bond in each mol­ecule with an *S*(6) ring motif. There are also short intra­molecular N—H⋯N and C—H⋯S contacts in each mol­ecule. In the crystal, mol­ecules are linked *via* C—H⋯S hydrogen bonds and C—H⋯π inter­actions, forming a three-dimensional structure. The crystal was refined as a non-merohedral twin with a final BASF value of 0.110 (1).

## Related literature   

For the biological importance of thio­semicarbazones, see: Chellan *et al.* (2010 ▸); Prabhakaran *et al.* (2008 ▸); Kelly *et al.* (1996 ▸). For binding motifs of thio­semicarbazones, see: Lobana *et al.* (2009 ▸). For thio­semicarbazones as ligands in catalysis, see: Xie *et al.* (2010 ▸). For related structures, see: Qasem Ali *et al.* (2011 ▸); Ramzan *et al.* (2010 ▸).
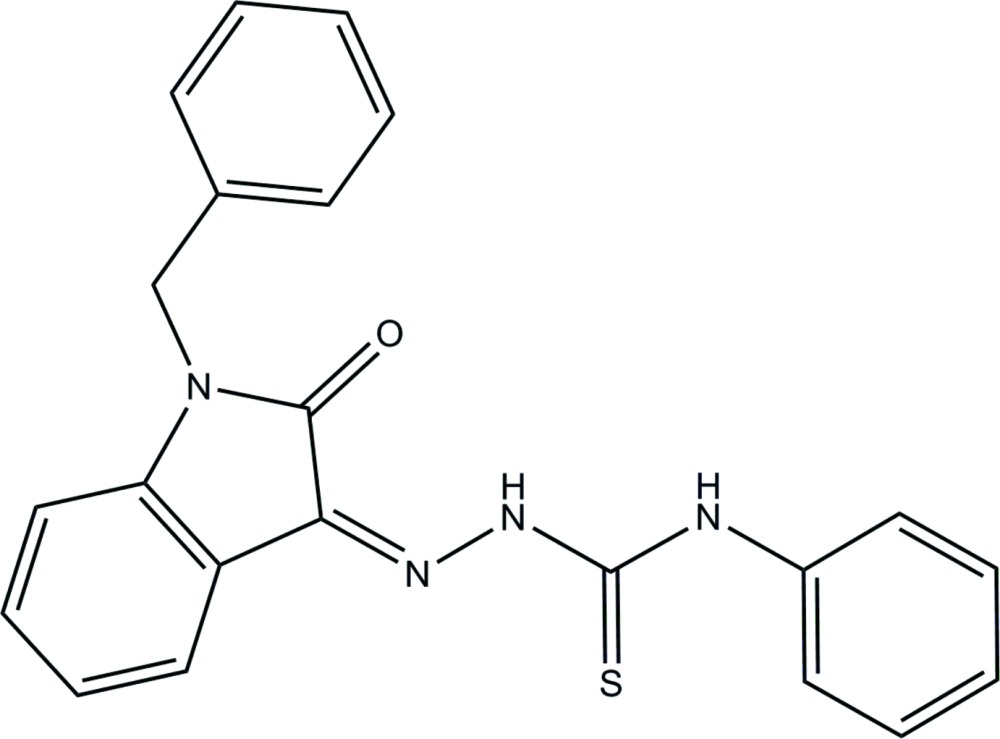


## Experimental   

### Crystal data  

C_22_H_18_N_4_OS
*M* *_r_* = 386.46
Triclinic, 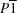

*a* = 11.2426 (3) Å
*b* = 11.4899 (3) Å
*c* = 30.3720 (9) Å
α = 79.121 (1)°
β = 88.628 (2)°
γ = 81.850 (1)°
*V* = 3813.91 (18) Å^3^
*Z* = 8
Mo *K*α radiation
μ = 0.19 mm^−1^
*T* = 293 K
0.35 × 0.30 × 0.30 mm

### Data collection  

Bruker Kappa APEXII CCD diffractometer
Absorption correction: multi-scan (*SADABS*; Bruker, 2004 ▸) *T*_min_ = 0.936, *T*_max_ = 0.945
66921 measured reflections
13397 independent reflections
5697 reflections with *I* > 2σ(*I*)
*R*_int_ = 0.086

### Refinement  

*R*[*F* ^2^ > 2σ(*F* ^2^)] = 0.053
*wR*(*F* ^2^) = 0.156
*S* = 0.99
13397 reflections
1011 parameters
72 restraints
H-atom parameters constrained
Δρ_max_ = 0.24 e Å^−3^
Δρ_min_ = −0.26 e Å^−3^

### 

Data collection: *APEX2* (Bruker, 2004 ▸); cell refinement: *APEX2* and *SAINT* (Bruker, 2004 ▸); data reduction: *SAINT* and *XPREP* (Bruker, 2004 ▸); program(s) used to solve structure: *SIR92* (Altomare *et al.*, 1993 ▸); program(s) used to refine structure: *SHELXL97* (Sheldrick, 2008 ▸); molecular graphics: *PLATON* (Spek, 2009 ▸); software used to prepare material for publication: *SHELXL97* and *PLATON*.

## Supplementary Material

Crystal structure: contains datablock(s) global, I. DOI: 10.1107/S2056989015002248/su5069sup1.cif

Structure factors: contains datablock(s) I. DOI: 10.1107/S2056989015002248/su5069Isup2.hkl

Supporting information file. DOI: 10.1107/S2056989015002248/su5069Isup3.cml

**Fig. 1.**
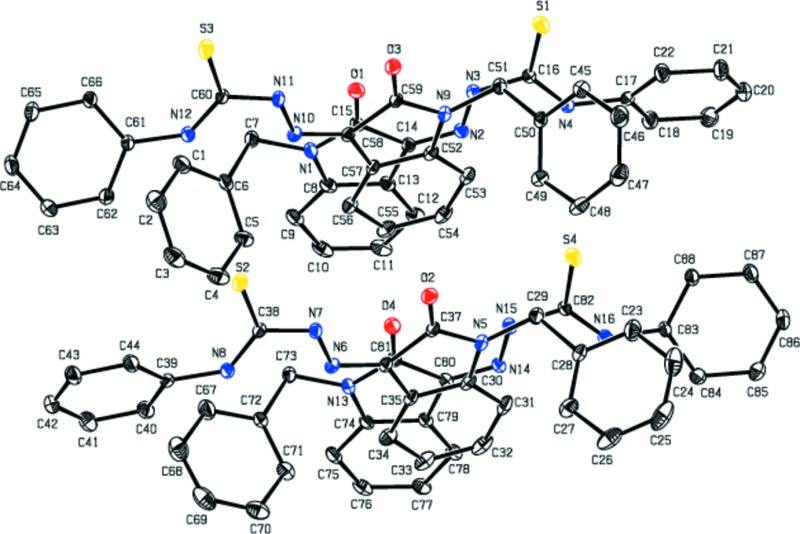
The molecular structure of the four independent molecules (atom S1 is in molecule A, S2 is in B, S3 in C and S4 in D) of the title compound, with atom labelling. Displacement ellipsoids are drawn at the 10% probability level.

**Fig. 2.**
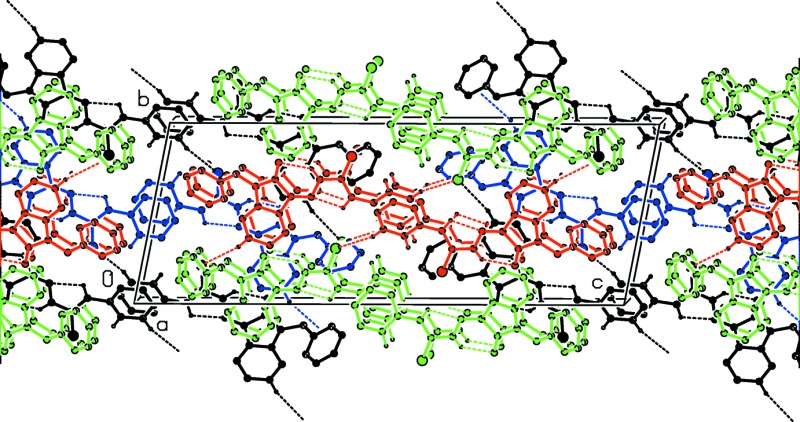
A view along the a axis of the crystal packing of the title compound showing the hydrogen bonds as dashed lines (see Table 1 for details; molecule colour code: A black, B red, C green, D blue). Hydrogen atoms not involved in hydrogen bonding have been omitted for clarity.

A B C D . DOI: 10.1107/S2056989015002248/su5069fig1.tif
The mol­ecular structure of the four independent mol­ecules (atom S1 is in mol­ecule *A*, S2 is in *B*, S3 in *C* and S4 in *D*) of the title compound, with atom labelling. Displacement ellipsoids are drawn at the 10% probability level.

a A B C D . DOI: 10.1107/S2056989015002248/su5069fig2.tif
A view along the *a* axis of the crystal packing of the title compound showing the hydrogen bonds as dashed lines (see Table 1 for details; mol­ecule colour code: *A* black, *B* red, *C* green, *D* blue). Hydrogen atoms not involved in hydrogen bonding have been omitted for clarity.

CCDC reference: 1046916

Additional supporting information:  crystallographic information; 3D view; checkCIF report

## Figures and Tables

**Table 1:** Hydrogen-bond geometry (, ) *Cg*2 and *Cg*12 are the centroids of rings C1C6 (molecule *A*) and C45C50 (molecule *C*), respectively.

*D*H*A*	*D*H	H*A*	*D* *A*	*D*H*A*
N3H3*A*O1	0.86	2.11	2.777(4)	134
N7H7O2	0.86	2.10	2.773(4)	135
N11H11*A*O3	0.86	2.09	2.771(4)	135
N15H15O4	0.86	2.10	2.779(4)	135
N4H4*A*N2	0.86	2.13	2.575(4)	112
N8H8N6	0.86	2.12	2.570(5)	112
N12H12*A*N10	0.86	2.17	2.606(4)	111
N16H16N14	0.86	2.16	2.603(4)	111
C22H22S1	0.93	2.58	3.203(5)	125
C44H44S2	0.93	2.61	3.224(5)	124
C66H66S3	0.93	2.67	3.217(4)	119
C88H88S4	0.93	2.67	3.227(4)	120
C10H10S3^i^	0.93	2.80	3.697(5)	164
C21H21S4^ii^	0.93	2.80	3.710(5)	166
C32H32S1^iii^	0.93	2.74	3.648(6)	166
C43H43S3^iv^	0.93	2.81	3.712(6)	165
C76H76S2^i^	0.93	2.84	3.721(5)	159
C41H41*Cg*2^v^	0.93	2.99	3.788(6)	145
C78H78*Cg*12^i^	0.93	2.95	3.717(5)	141

Symmetry codes: (i) 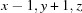; (ii) 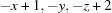; (iii) 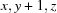; (iv) 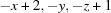; (v) 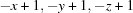.

## supplementary crystallographic information

### S1. Synthesis and crystallization

*N*-phenyl­hydrazine carbo­thio­amide (1.65 g; 0.01 mol) was dissolved in 20 ml of hot ethanol and to this 2.31 g of (0.01 mol) 1-benzyl­indoline-2,3-dionein 10 ml of ethanol was added over a period of 10 min with continuous sirring. The reaction mixture was refluxed for 1 h and the mixture was then allowed to cool to room temperature, whereby a shinny yellow compound began to separate. It was filtered off and washed with ethanol and dried under vacuum. The compound was recrystallized from hot ethanol (yield: 89%). Single crystals suitable for X-ray diffraction were obtained by slow evaporation of a solution of the title compound in ethanol at room temperature.

### S2. Refinement

Crystal data, data collection and structure refinement details are summarized in Table 2. The NH H atoms and the C-bound H atoms were fixed geometrically and allowed to ride on their parent atoms: N—H = 0.86 Å and C—H = 0.93–0.97 Å with *U*_iso_(H) = 1.5*U*_eq_(C) for methyl H atoms and = 1.2*U*_eq_(N,C) for other H atoms. The crystal was refined as a non-merohedral twin [matrix: 0 0 -1, 0 -1 0 0 -1 1] with a final BASF value of 0.110 (1).

### S3. Comment

The design and synthesis of thiosemicarbazones are of considerable interest because of their versatile chemistry and various biological activities such as antitumor, antibacterial, antiviral, antiamoebic and antimalarial (Kelly *et al.*, 1996). They comprise an intriguing class of chelating molecules which possess a wide range of beneficial medicinal properties (Prabhakaran *et al.*, 2008). Thiosemicarbazones are a versatile class of ligands that have been studied for their biological activity (Chellan *et al.*, 2010), their interesting binding motifs (Lobana *et al.*, 2009) and they use as ligands in catalysis (Xie *et al.*, 2010). In view of this biological importance, the crystal structure of the title compound has been determined and the results are presented herein.

The molecular structures of the four independent molecules (A_S1, B_S2, C_S3, D_S4) of the title compound are illustrated in Fig. 1. All four molecules have a Z conformation about the C═N bond with the benzyl ring being inclined to the indoline ring mean plane by 73.4 (2), 77.9 (2), 73.2 (2) and 77.2 (2) ° in molecules A, B, C and D, respectively. In molecules A and B the phenyl ring is inclined to the mean plane of the indoline ring mean plane by 12.0 (2) and 12.2 (2) °, respectively. However, in molecules C and D the same dihedral angles are larger; 37.3 (2) and 36.4 (2) °, respectively. Consequently, the benzyl and phenyl rings are almost normal to one another in molecules A and B (dihedral angles of 80.3 (3) and 87.1 (3) °, respectively), while in molecules C and D the same dihedral angles are only 48.8 (2) and 43.8 (3) °, respectively. There is an intramolecular N-H···O hydrogen bond in each molecule with an S(6) ring motif (Table 1).

In the crystal, molecules are linked via C-H···S hydrogen bonds and C-H···π interactions forming a three-dimensional structure (Table 1 and Fig. 2).

### Crystal data

**Table:**

C_22_H_18_N_4_OS	*Z* = 8
*M**_r_* = 386.46	*F*(000) = 1616
Triclinic, *P*1	*D*_x_ = 1.346 Mg m^−^^3^
Hall symbol: -P 1	Mo *K*α radiation, λ = 0.71073 Å
*a* = 11.2426 (3) Å	Cell parameters from 6770 reflections
*b* = 11.4899 (3) Å	θ = 2.3–26.5°
*c* = 30.3720 (9) Å	µ = 0.19 mm^−^^1^
α = 79.121 (1)°	*T* = 293 K
β = 88.628 (2)°	Block, colourless
γ = 81.850 (1)°	0.35 × 0.30 × 0.30 mm
*V* = 3813.91 (18) Å^3^	

### Data collection

**Table:**

Bruker Kappa APEXII CCD diffractometer	13397 independent reflections
Radiation source: fine-focus sealed tube	5697 reflections with *I* > 2σ(*I*)
Graphite monochromator	*R*_int_ = 0.086
ω and φ scan	θ_max_ = 25.0°, θ_min_ = 1.8°
Absorption correction: multi-scan (*SADABS*; Bruker, 2004)	*h* = −13→13
*T*_min_ = 0.936, *T*_max_ = 0.945	*k* = −13→13
66921 measured reflections	*l* = −36→36

### Refinement

**Table:**

Refinement on *F*^2^	Secondary atom site location: difference Fourier map
Least-squares matrix: full	Hydrogen site location: inferred from neighbouring sites
*R*[*F*^2^ > 2σ(*F*^2^)] = 0.053	H-atom parameters constrained
*wR*(*F*^2^) = 0.156	*w* = 1/[σ^2^(*F*_o_^2^) + (0.0578*P*)^2^] where *P* = (*F*_o_^2^ + 2*F*_c_^2^)/3
*S* = 0.99	(Δ/σ)_max_ = 0.001
13397 reflections	Δρ_max_ = 0.24 e Å^−^^3^
1011 parameters	Δρ_min_ = −0.26 e Å^−^^3^
72 restraints	Extinction correction: *SHELXL97* (Sheldrick, 2008), Fc^*^=kFc[1+0.001xFc^2^λ^3^/sin(2θ)]^-1/4^
Primary atom site location: structure-invariant direct methods	Extinction coefficient: 0.00065 (18)

### Special details

**Table:**

Geometry. All e.s.d.'s (except the e.s.d. in the dihedral angle between two l.s. planes) are estimated using the full covariance matrix. The cell e.s.d.'s are taken into account individually in the estimation of e.s.d.'s in distances, angles and torsion angles; correlations between e.s.d.'s in cell parameters are only used when they are defined by crystal symmetry. An approximate (isotropic) treatment of cell e.s.d.'s is used for estimating e.s.d.'s involving l.s. planes.
Refinement. Refinement of *F*^2^ against ALL reflections. The weighted *R*-factor *wR* and goodness of fit *S* are based on *F*^2^, conventional *R*-factors *R* are based on *F*, with *F* set to zero for negative *F*^2^. The threshold expression of *F*^2^ > σ(*F*^2^) is used only for calculating *R*-factors(gt) *etc*. and is not relevant to the choice of reflections for refinement. *R*-factors based on *F*^2^ are statistically about twice as large as those based on *F*, and *R*- factors based on ALL data will be even larger.

### Fractional atomic coordinates and isotropic or equivalent isotropic displacement parameters (Å^2^)

**Table:**

	*x*	*y*	*z*	*U*_iso_*/*U*_eq_	
C1	0.4308 (5)	0.1425 (4)	0.59335 (16)	0.0730 (15)	
H1	0.4028	0.0746	0.5875	0.088*	
C2	0.5234 (5)	0.1865 (5)	0.56815 (18)	0.0925 (18)	
H2	0.5582	0.1481	0.5457	0.111*	
C3	0.5641 (5)	0.2871 (6)	0.5763 (2)	0.0974 (19)	
H3	0.6265	0.3174	0.5593	0.117*	
C4	0.5134 (5)	0.3432 (4)	0.6094 (2)	0.0840 (17)	
H4	0.5410	0.4117	0.6149	0.101*	
C5	0.4210 (4)	0.2976 (4)	0.63481 (15)	0.0659 (14)	
H5	0.3868	0.3356	0.6575	0.079*	
C6	0.3792 (4)	0.1967 (4)	0.62687 (14)	0.0550 (12)	
C7	0.2791 (4)	0.1416 (4)	0.65306 (13)	0.0606 (13)	
H7A	0.2046	0.1706	0.6366	0.073*	
H7B	0.2945	0.0556	0.6549	0.073*	
C8	0.1921 (4)	0.2667 (4)	0.71005 (15)	0.0537 (12)	
C9	0.1293 (4)	0.3636 (4)	0.68257 (16)	0.0665 (14)	
H9	0.1285	0.3693	0.6516	0.080*	
C10	0.0681 (4)	0.4515 (4)	0.70247 (19)	0.0719 (15)	
H10	0.0256	0.5185	0.6846	0.086*	
C11	0.0681 (4)	0.4433 (4)	0.74804 (18)	0.0696 (14)	
H11	0.0255	0.5047	0.7605	0.084*	
C12	0.1304 (4)	0.3452 (4)	0.77597 (15)	0.0589 (13)	
H12	0.1306	0.3396	0.8069	0.071*	
C13	0.1917 (4)	0.2568 (4)	0.75615 (14)	0.0481 (11)	
C14	0.2628 (4)	0.1443 (3)	0.77424 (14)	0.0446 (11)	
C15	0.3084 (4)	0.0874 (4)	0.73554 (14)	0.0494 (12)	
C16	0.3670 (4)	−0.0559 (3)	0.87169 (13)	0.0448 (11)	
C17	0.3236 (4)	−0.0010 (3)	0.94674 (13)	0.0482 (12)	
C18	0.2405 (5)	0.0756 (4)	0.96406 (15)	0.0721 (15)	
H18	0.1903	0.1339	0.9450	0.087*	
C19	0.2301 (5)	0.0670 (5)	1.01031 (19)	0.0894 (18)	
H19	0.1739	0.1201	1.0222	0.107*	
C20	0.3022 (6)	−0.0190 (5)	1.03778 (17)	0.093 (2)	
H20	0.2957	−0.0254	1.0687	0.112*	
C21	0.3837 (5)	−0.0955 (5)	1.02036 (16)	0.0872 (18)	
H21	0.4330	−0.1547	1.0395	0.105*	
C22	0.3951 (5)	−0.0873 (4)	0.97432 (15)	0.0731 (15)	
H22	0.4513	−0.1407	0.9626	0.088*	
C23	0.8843 (5)	0.2667 (5)	0.91713 (18)	0.0883 (17)	
H23	0.8416	0.2037	0.9280	0.106*	
C24	0.9653 (7)	0.2974 (7)	0.9441 (2)	0.122 (3)	
H24	0.9759	0.2560	0.9734	0.147*	
C25	1.0303 (7)	0.3868 (7)	0.9289 (3)	0.127 (3)	
H25	1.0868	0.4057	0.9473	0.153*	
C26	1.0119 (5)	0.4485 (5)	0.8865 (2)	0.102 (2)	
H26	1.0562	0.5103	0.8757	0.122*	
C27	0.9285 (5)	0.4204 (5)	0.85956 (17)	0.0764 (15)	
H27	0.9148	0.4651	0.8308	0.092*	
C28	0.8655 (4)	0.3277 (4)	0.87440 (15)	0.0551 (12)	
C29	0.7754 (4)	0.2896 (4)	0.84633 (13)	0.0650 (13)	
H29A	0.7998	0.2062	0.8445	0.078*	
H29B	0.6983	0.2952	0.8614	0.078*	
C30	0.6915 (4)	0.4712 (4)	0.78936 (15)	0.0527 (12)	
C31	0.6287 (5)	0.5411 (5)	0.81688 (15)	0.0692 (14)	
H31	0.6255	0.5147	0.8477	0.083*	
C32	0.5713 (5)	0.6508 (5)	0.7969 (2)	0.0784 (16)	
H32	0.5299	0.7006	0.8147	0.094*	
C33	0.5732 (4)	0.6895 (4)	0.75107 (19)	0.0729 (15)	
H33	0.5332	0.7646	0.7386	0.087*	
C34	0.6338 (4)	0.6183 (4)	0.72355 (16)	0.0615 (13)	
H34	0.6345	0.6435	0.6926	0.074*	
C35	0.6929 (4)	0.5091 (4)	0.74348 (14)	0.0483 (11)	
C36	0.7632 (4)	0.4136 (3)	0.72551 (14)	0.0441 (11)	
C37	0.8055 (4)	0.3168 (4)	0.76421 (14)	0.0477 (11)	
C38	0.8705 (4)	0.3113 (3)	0.62843 (13)	0.0442 (11)	
C39	0.8292 (4)	0.4437 (3)	0.55339 (13)	0.0456 (11)	
C40	0.7483 (5)	0.5406 (4)	0.53586 (16)	0.0717 (15)	
H40	0.7012	0.5825	0.5549	0.086*	
C41	0.7362 (5)	0.5762 (4)	0.49040 (19)	0.0921 (18)	
H41	0.6802	0.6416	0.4786	0.110*	
C42	0.8067 (6)	0.5153 (5)	0.46216 (17)	0.0877 (18)	
H42	0.7998	0.5398	0.4312	0.105*	
C43	0.8859 (5)	0.4197 (5)	0.47991 (17)	0.0858 (18)	
H43	0.9324	0.3774	0.4609	0.103*	
C44	0.8996 (5)	0.3830 (4)	0.52544 (15)	0.0683 (14)	
H44	0.9561	0.3178	0.5371	0.082*	
C45	0.9260 (5)	−0.2562 (4)	0.89871 (16)	0.0718 (15)	
H45	0.8977	−0.3298	0.9042	0.086*	
C46	1.0188 (5)	−0.2397 (6)	0.92375 (17)	0.0920 (18)	
H46	1.0545	−0.3023	0.9454	0.110*	
C47	1.0595 (5)	−0.1317 (6)	0.91714 (18)	0.0905 (18)	
H47	1.1216	−0.1197	0.9347	0.109*	
C48	1.0087 (5)	−0.0414 (5)	0.88459 (19)	0.0784 (16)	
H48	1.0366	0.0323	0.8798	0.094*	
C49	0.9157 (4)	−0.0588 (4)	0.85858 (15)	0.0590 (12)	
H49	0.8819	0.0032	0.8363	0.071*	
C50	0.8730 (4)	−0.1665 (4)	0.86547 (13)	0.0493 (11)	
C51	0.7730 (4)	−0.1932 (4)	0.83898 (13)	0.0575 (12)	
H51A	0.7868	−0.2774	0.8371	0.069*	
H51B	0.6983	−0.1791	0.8550	0.069*	
C52	0.6883 (4)	−0.0110 (4)	0.78161 (14)	0.0480 (11)	
C53	0.6259 (4)	0.0589 (4)	0.80913 (15)	0.0581 (13)	
H53	0.6252	0.0342	0.8401	0.070*	
C54	0.5644 (4)	0.1675 (4)	0.78837 (17)	0.0643 (14)	
H54	0.5214	0.2167	0.8060	0.077*	
C55	0.5645 (4)	0.2051 (4)	0.74273 (17)	0.0623 (13)	
H55	0.5226	0.2792	0.7301	0.075*	
C56	0.6266 (4)	0.1337 (4)	0.71538 (14)	0.0535 (12)	
H56	0.6262	0.1582	0.6844	0.064*	
C57	0.6888 (4)	0.0256 (4)	0.73531 (13)	0.0433 (11)	
C58	0.7596 (4)	−0.0699 (3)	0.71757 (14)	0.0431 (11)	
C59	0.8039 (4)	−0.1651 (4)	0.75693 (13)	0.0465 (11)	
C60	0.8641 (4)	−0.1789 (3)	0.62138 (13)	0.0438 (11)	
C61	0.8310 (4)	−0.0578 (3)	0.54467 (13)	0.0451 (11)	
C62	0.8337 (4)	0.0574 (4)	0.52315 (15)	0.0685 (14)	
H62	0.8338	0.1171	0.5400	0.082*	
C63	0.8365 (5)	0.0871 (4)	0.47749 (16)	0.0807 (16)	
H63	0.8400	0.1658	0.4635	0.097*	
C64	0.8339 (5)	0.0012 (5)	0.45270 (15)	0.0757 (15)	
H64	0.8343	0.0210	0.4216	0.091*	
C65	0.8308 (4)	−0.1137 (4)	0.47322 (14)	0.0689 (14)	
H65	0.8305	−0.1727	0.4561	0.083*	
C66	0.8281 (4)	−0.1441 (4)	0.51944 (13)	0.0596 (13)	
H66	0.8243	−0.2229	0.5333	0.071*	
C67	0.3862 (5)	0.6784 (4)	0.59113 (17)	0.0817 (16)	
H67	0.3455	0.6231	0.5812	0.098*	
C68	0.4691 (6)	0.7320 (6)	0.56366 (19)	0.105 (2)	
H68	0.4823	0.7148	0.5351	0.126*	
C69	0.5310 (6)	0.8090 (6)	0.5779 (2)	0.105 (2)	
H69	0.5893	0.8431	0.5596	0.126*	
C70	0.5094 (5)	0.8373 (4)	0.6184 (2)	0.0902 (17)	
H70	0.5522	0.8911	0.6282	0.108*	
C71	0.4224 (4)	0.7856 (4)	0.64582 (15)	0.0671 (13)	
H71	0.4054	0.8071	0.6736	0.080*	
C72	0.3621 (4)	0.7039 (4)	0.63229 (14)	0.0497 (11)	
C73	0.2704 (4)	0.6419 (4)	0.66060 (13)	0.0596 (13)	
H73A	0.1929	0.6662	0.6459	0.072*	
H73B	0.2909	0.5564	0.6621	0.072*	
C74	0.1903 (4)	0.7660 (4)	0.71799 (14)	0.0501 (12)	
C75	0.1282 (4)	0.8646 (4)	0.69089 (15)	0.0629 (14)	
H75	0.1259	0.8712	0.6599	0.076*	
C76	0.0699 (4)	0.9528 (4)	0.71167 (17)	0.0665 (14)	
H76	0.0280	1.0206	0.6941	0.080*	
C77	0.0710 (4)	0.9446 (4)	0.75736 (16)	0.0619 (13)	
H77	0.0309	1.0064	0.7702	0.074*	
C78	0.1319 (4)	0.8444 (4)	0.78415 (14)	0.0550 (12)	
H78	0.1327	0.8372	0.8152	0.066*	
C79	0.1913 (4)	0.7556 (3)	0.76387 (13)	0.0448 (11)	
C80	0.2605 (4)	0.6412 (3)	0.78177 (13)	0.0443 (11)	
C81	0.3030 (4)	0.5853 (4)	0.74258 (14)	0.0482 (11)	
C82	0.3649 (4)	0.4344 (3)	0.87802 (13)	0.0447 (11)	
C83	0.3328 (4)	0.4814 (3)	0.95437 (13)	0.0468 (11)	
C84	0.3363 (4)	0.5760 (4)	0.97534 (15)	0.0716 (15)	
H84	0.3378	0.6521	0.9584	0.086*	
C85	0.3376 (5)	0.5592 (5)	1.02159 (17)	0.0862 (17)	
H85	0.3410	0.6238	1.0357	0.103*	
C86	0.3339 (5)	0.4479 (5)	1.04647 (15)	0.0787 (16)	
H86	0.3334	0.4367	1.0776	0.094*	
C87	0.3311 (4)	0.3540 (4)	1.02593 (15)	0.0725 (15)	
H87	0.3301	0.2780	1.0431	0.087*	
C88	0.3296 (4)	0.3690 (4)	0.98011 (13)	0.0605 (13)	
H88	0.3264	0.3038	0.9664	0.073*	
N1	0.2633 (3)	0.1661 (3)	0.69810 (11)	0.0541 (10)	
N2	0.2796 (3)	0.1017 (3)	0.81601 (11)	0.0464 (9)	
N3	0.3455 (3)	−0.0072 (3)	0.82758 (10)	0.0472 (9)	
H3A	0.3739	−0.0458	0.8071	0.057*	
N4	0.3272 (3)	0.0179 (3)	0.89942 (10)	0.0508 (9)	
H4A	0.2990	0.0892	0.8863	0.061*	
N5	0.7603 (3)	0.3580 (3)	0.80158 (11)	0.0556 (10)	
N6	0.7811 (3)	0.4142 (3)	0.68355 (11)	0.0465 (9)	
N7	0.8455 (3)	0.3161 (3)	0.67240 (10)	0.0466 (9)	
H7	0.8710	0.2561	0.6930	0.056*	
N8	0.8344 (3)	0.4142 (3)	0.60078 (11)	0.0530 (10)	
H8	0.8099	0.4730	0.6140	0.064*	
N9	0.7595 (3)	−0.1233 (3)	0.79404 (11)	0.0513 (9)	
N10	0.7764 (3)	−0.0716 (3)	0.67580 (11)	0.0457 (9)	
N11	0.8413 (3)	−0.1703 (3)	0.66506 (10)	0.0481 (9)	
H11A	0.8688	−0.2286	0.6860	0.058*	
N12	0.8267 (3)	−0.0803 (3)	0.59219 (10)	0.0484 (9)	
H12A	0.7948	−0.0206	0.6038	0.058*	
N13	0.2590 (3)	0.6647 (3)	0.70560 (11)	0.0526 (10)	
N14	0.2770 (3)	0.5973 (3)	0.82362 (10)	0.0461 (9)	
N15	0.3411 (3)	0.4869 (3)	0.83436 (10)	0.0494 (9)	
H15	0.3672	0.4493	0.8134	0.059*	
N16	0.3292 (3)	0.5056 (3)	0.90713 (10)	0.0515 (9)	
H16	0.2993	0.5775	0.8953	0.062*	
O1	0.3709 (3)	−0.0088 (2)	0.73653 (9)	0.0587 (8)	
O2	0.8680 (3)	0.2219 (2)	0.76334 (9)	0.0579 (8)	
O3	0.8657 (3)	−0.2600 (2)	0.75620 (9)	0.0569 (8)	
O4	0.3647 (3)	0.4898 (2)	0.74290 (9)	0.0577 (8)	
S1	0.43742 (12)	−0.19389 (9)	0.88417 (4)	0.0692 (4)	
S2	0.93895 (12)	0.18469 (9)	0.61623 (4)	0.0623 (4)	
S3	0.93619 (13)	−0.30667 (10)	0.61162 (4)	0.0708 (4)	
S4	0.43512 (12)	0.29630 (9)	0.88779 (4)	0.0675 (4)	

### Atomic displacement parameters (Å^2^)

**Table:**

	*U*^11^	*U*^22^	*U*^33^	*U*^12^	*U*^13^	*U*^23^
C1	0.075 (4)	0.076 (3)	0.066 (4)	−0.021 (3)	0.002 (3)	0.000 (3)
C2	0.086 (5)	0.105 (5)	0.084 (4)	−0.012 (4)	0.022 (4)	−0.015 (3)
C3	0.079 (5)	0.106 (5)	0.098 (5)	−0.029 (4)	0.015 (4)	0.013 (4)
C4	0.066 (4)	0.073 (4)	0.108 (5)	−0.022 (3)	−0.004 (4)	0.007 (3)
C5	0.055 (4)	0.060 (3)	0.079 (4)	−0.016 (3)	−0.005 (3)	0.003 (3)
C6	0.056 (3)	0.061 (3)	0.043 (3)	−0.011 (3)	−0.006 (2)	0.007 (2)
C7	0.067 (4)	0.077 (3)	0.042 (3)	−0.030 (3)	−0.004 (2)	−0.007 (2)
C8	0.042 (3)	0.054 (3)	0.061 (3)	−0.013 (2)	−0.009 (2)	0.006 (3)
C9	0.058 (4)	0.070 (3)	0.064 (3)	−0.022 (3)	−0.015 (3)	0.018 (3)
C10	0.050 (4)	0.057 (3)	0.092 (4)	−0.006 (3)	−0.007 (3)	0.026 (3)
C11	0.048 (4)	0.053 (3)	0.099 (4)	0.001 (3)	0.007 (3)	0.002 (3)
C12	0.050 (3)	0.048 (3)	0.076 (3)	−0.006 (2)	−0.001 (3)	−0.003 (3)
C13	0.042 (3)	0.048 (3)	0.051 (3)	−0.008 (2)	−0.008 (2)	0.003 (2)
C14	0.044 (3)	0.049 (3)	0.041 (3)	−0.012 (2)	−0.001 (2)	−0.005 (2)
C15	0.052 (3)	0.053 (3)	0.044 (3)	−0.021 (3)	−0.002 (2)	0.001 (2)
C16	0.051 (3)	0.043 (2)	0.040 (3)	−0.008 (2)	0.002 (2)	−0.005 (2)
C17	0.063 (4)	0.041 (2)	0.043 (3)	−0.009 (2)	0.003 (2)	−0.013 (2)
C18	0.101 (5)	0.058 (3)	0.055 (3)	0.002 (3)	0.002 (3)	−0.016 (2)
C19	0.118 (6)	0.082 (4)	0.075 (4)	−0.012 (4)	0.027 (4)	−0.035 (3)
C20	0.149 (7)	0.087 (4)	0.049 (4)	−0.036 (4)	0.013 (4)	−0.015 (3)
C21	0.127 (6)	0.081 (4)	0.048 (4)	0.001 (4)	−0.016 (3)	−0.008 (3)
C22	0.101 (5)	0.061 (3)	0.051 (3)	0.008 (3)	−0.008 (3)	−0.007 (2)
C23	0.099 (5)	0.099 (4)	0.066 (4)	−0.006 (4)	−0.020 (3)	−0.017 (3)
C24	0.115 (7)	0.168 (7)	0.079 (5)	0.012 (5)	−0.032 (5)	−0.030 (5)
C25	0.083 (6)	0.174 (8)	0.139 (8)	0.025 (5)	−0.060 (6)	−0.089 (7)
C26	0.053 (4)	0.124 (5)	0.152 (6)	−0.022 (4)	−0.002 (4)	−0.079 (5)
C27	0.065 (4)	0.092 (4)	0.083 (4)	−0.018 (3)	0.000 (3)	−0.036 (3)
C28	0.056 (4)	0.065 (3)	0.049 (3)	−0.006 (3)	0.000 (3)	−0.025 (2)
C29	0.078 (4)	0.077 (3)	0.047 (3)	−0.032 (3)	0.003 (3)	−0.015 (2)
C30	0.048 (3)	0.063 (3)	0.057 (3)	−0.017 (3)	0.004 (3)	−0.033 (3)
C31	0.068 (4)	0.098 (4)	0.056 (3)	−0.023 (3)	0.007 (3)	−0.044 (3)
C32	0.053 (4)	0.096 (4)	0.107 (5)	−0.013 (3)	0.006 (3)	−0.068 (4)
C33	0.054 (4)	0.076 (3)	0.099 (4)	−0.001 (3)	−0.009 (3)	−0.048 (3)
C34	0.051 (3)	0.061 (3)	0.076 (3)	0.001 (3)	−0.003 (3)	−0.029 (3)
C35	0.046 (3)	0.058 (3)	0.047 (3)	−0.008 (2)	0.004 (2)	−0.026 (2)
C36	0.044 (3)	0.051 (3)	0.041 (3)	−0.008 (2)	0.003 (2)	−0.018 (2)
C37	0.049 (3)	0.058 (3)	0.043 (3)	−0.015 (3)	0.002 (2)	−0.021 (2)
C38	0.046 (3)	0.046 (3)	0.042 (3)	−0.005 (2)	−0.005 (2)	−0.014 (2)
C39	0.055 (3)	0.044 (2)	0.041 (3)	−0.013 (2)	0.005 (2)	−0.013 (2)
C40	0.095 (4)	0.051 (3)	0.062 (3)	0.003 (3)	−0.005 (3)	−0.003 (2)
C41	0.120 (6)	0.072 (4)	0.073 (4)	−0.004 (4)	−0.024 (4)	0.011 (3)
C42	0.136 (6)	0.082 (4)	0.047 (3)	−0.038 (4)	−0.007 (4)	0.001 (3)
C43	0.129 (6)	0.073 (4)	0.054 (4)	−0.017 (4)	0.022 (3)	−0.010 (3)
C44	0.091 (4)	0.058 (3)	0.053 (3)	−0.005 (3)	0.008 (3)	−0.007 (3)
C45	0.081 (4)	0.072 (3)	0.064 (3)	−0.020 (3)	−0.005 (3)	−0.011 (3)
C46	0.091 (5)	0.109 (5)	0.074 (4)	−0.011 (4)	−0.023 (3)	−0.009 (3)
C47	0.078 (5)	0.122 (5)	0.081 (4)	−0.023 (4)	−0.017 (3)	−0.033 (4)
C48	0.071 (4)	0.086 (4)	0.095 (4)	−0.027 (3)	0.006 (3)	−0.048 (3)
C49	0.057 (4)	0.058 (3)	0.066 (3)	−0.010 (3)	0.002 (3)	−0.021 (2)
C50	0.051 (3)	0.060 (3)	0.040 (3)	−0.008 (3)	0.007 (2)	−0.018 (2)
C51	0.068 (4)	0.067 (3)	0.041 (3)	−0.021 (3)	0.002 (2)	−0.010 (2)
C52	0.041 (3)	0.057 (3)	0.053 (3)	−0.012 (2)	0.002 (2)	−0.024 (2)
C53	0.052 (3)	0.077 (3)	0.054 (3)	−0.017 (3)	0.003 (3)	−0.031 (3)
C54	0.045 (3)	0.085 (4)	0.077 (4)	−0.013 (3)	0.008 (3)	−0.049 (3)
C55	0.052 (4)	0.062 (3)	0.079 (4)	−0.005 (3)	0.000 (3)	−0.032 (3)
C56	0.054 (3)	0.055 (3)	0.054 (3)	−0.004 (2)	−0.003 (2)	−0.019 (2)
C57	0.040 (3)	0.055 (3)	0.040 (3)	−0.012 (2)	0.001 (2)	−0.019 (2)
C58	0.041 (3)	0.051 (3)	0.042 (3)	−0.013 (2)	−0.001 (2)	−0.014 (2)
C59	0.050 (3)	0.055 (3)	0.040 (3)	−0.017 (3)	0.002 (2)	−0.015 (2)
C60	0.048 (3)	0.047 (3)	0.038 (3)	−0.005 (2)	−0.001 (2)	−0.013 (2)
C61	0.049 (3)	0.048 (3)	0.037 (3)	−0.001 (2)	0.000 (2)	−0.008 (2)
C62	0.103 (5)	0.050 (3)	0.052 (3)	−0.007 (3)	−0.016 (3)	−0.009 (2)
C63	0.117 (5)	0.063 (3)	0.059 (4)	−0.023 (3)	−0.013 (3)	0.006 (3)
C64	0.091 (4)	0.089 (4)	0.043 (3)	−0.014 (3)	−0.008 (3)	−0.001 (3)
C65	0.084 (4)	0.078 (4)	0.045 (3)	−0.001 (3)	−0.007 (3)	−0.019 (3)
C66	0.085 (4)	0.051 (3)	0.042 (3)	−0.003 (3)	0.003 (2)	−0.013 (2)
C67	0.094 (4)	0.088 (3)	0.060 (3)	−0.013 (3)	0.013 (3)	−0.008 (3)
C68	0.108 (5)	0.122 (4)	0.078 (4)	0.002 (4)	0.034 (4)	−0.018 (3)
C69	0.083 (5)	0.111 (4)	0.101 (5)	−0.003 (4)	0.032 (4)	0.018 (4)
C70	0.068 (4)	0.076 (3)	0.118 (4)	−0.012 (3)	0.001 (4)	0.007 (3)
C71	0.061 (3)	0.071 (3)	0.064 (3)	−0.011 (3)	0.001 (3)	0.001 (2)
C72	0.053 (3)	0.050 (2)	0.043 (3)	−0.006 (2)	−0.002 (2)	0.001 (2)
C73	0.073 (4)	0.072 (3)	0.039 (3)	−0.028 (3)	0.000 (2)	−0.010 (2)
C74	0.052 (3)	0.049 (3)	0.045 (3)	−0.015 (2)	−0.006 (2)	0.006 (2)
C75	0.064 (4)	0.066 (3)	0.052 (3)	−0.019 (3)	−0.007 (3)	0.014 (3)
C76	0.049 (4)	0.058 (3)	0.080 (4)	−0.010 (3)	−0.007 (3)	0.020 (3)
C77	0.052 (4)	0.054 (3)	0.074 (4)	−0.004 (2)	0.003 (3)	−0.001 (3)
C78	0.054 (3)	0.052 (3)	0.055 (3)	−0.006 (2)	0.000 (2)	0.001 (2)
C79	0.041 (3)	0.046 (3)	0.045 (3)	−0.007 (2)	−0.002 (2)	0.001 (2)
C80	0.043 (3)	0.045 (2)	0.041 (3)	−0.007 (2)	0.001 (2)	−0.001 (2)
C81	0.049 (3)	0.050 (3)	0.046 (3)	−0.013 (2)	0.001 (2)	−0.005 (2)
C82	0.049 (3)	0.043 (2)	0.039 (3)	−0.005 (2)	−0.002 (2)	−0.001 (2)
C83	0.055 (3)	0.050 (3)	0.033 (2)	−0.005 (2)	0.002 (2)	−0.003 (2)
C84	0.104 (5)	0.055 (3)	0.058 (3)	−0.014 (3)	0.015 (3)	−0.014 (2)
C85	0.126 (5)	0.081 (4)	0.064 (4)	−0.027 (4)	0.012 (3)	−0.034 (3)
C86	0.101 (5)	0.097 (4)	0.039 (3)	−0.019 (4)	0.003 (3)	−0.012 (3)
C87	0.100 (5)	0.068 (3)	0.044 (3)	−0.005 (3)	0.006 (3)	−0.002 (3)
C88	0.083 (4)	0.056 (3)	0.040 (3)	−0.005 (3)	0.001 (2)	−0.007 (2)
N1	0.061 (3)	0.060 (2)	0.040 (2)	−0.017 (2)	−0.0018 (19)	−0.0018 (19)
N2	0.048 (3)	0.045 (2)	0.045 (2)	−0.0060 (18)	−0.0021 (18)	−0.0047 (17)
N3	0.054 (3)	0.043 (2)	0.041 (2)	0.0038 (18)	0.0023 (18)	−0.0082 (16)
N4	0.071 (3)	0.0370 (19)	0.042 (2)	0.0023 (18)	−0.0008 (19)	−0.0072 (17)
N5	0.072 (3)	0.061 (2)	0.040 (2)	−0.017 (2)	0.002 (2)	−0.0183 (19)
N6	0.049 (3)	0.046 (2)	0.048 (2)	−0.0016 (18)	0.0028 (18)	−0.0199 (17)
N7	0.055 (3)	0.047 (2)	0.037 (2)	−0.0015 (19)	0.0008 (17)	−0.0103 (16)
N8	0.075 (3)	0.042 (2)	0.043 (2)	−0.0019 (19)	0.0039 (19)	−0.0129 (17)
N9	0.061 (3)	0.057 (2)	0.038 (2)	−0.012 (2)	−0.0010 (19)	−0.0119 (18)
N10	0.047 (3)	0.048 (2)	0.045 (2)	−0.0009 (18)	0.0053 (18)	−0.0199 (17)
N11	0.060 (3)	0.048 (2)	0.035 (2)	0.0013 (19)	−0.0034 (18)	−0.0109 (16)
N12	0.060 (3)	0.042 (2)	0.042 (2)	0.0052 (18)	0.0044 (18)	−0.0128 (17)
N13	0.065 (3)	0.053 (2)	0.038 (2)	−0.010 (2)	0.0013 (19)	−0.0038 (18)
N14	0.050 (3)	0.043 (2)	0.041 (2)	−0.0048 (18)	−0.0024 (17)	0.0012 (16)
N15	0.057 (3)	0.044 (2)	0.042 (2)	0.0042 (18)	−0.0020 (18)	−0.0036 (17)
N16	0.067 (3)	0.0419 (19)	0.042 (2)	0.0006 (18)	−0.0021 (19)	−0.0048 (17)
O1	0.067 (2)	0.0501 (18)	0.059 (2)	−0.0077 (17)	0.0055 (17)	−0.0101 (15)
O2	0.064 (2)	0.0516 (18)	0.059 (2)	−0.0006 (17)	−0.0059 (16)	−0.0161 (15)
O3	0.063 (2)	0.0520 (18)	0.0554 (19)	−0.0046 (17)	−0.0037 (16)	−0.0118 (15)
O4	0.063 (2)	0.0520 (18)	0.0564 (19)	−0.0021 (17)	0.0033 (16)	−0.0104 (15)
S1	0.0962 (11)	0.0468 (7)	0.0547 (8)	0.0141 (7)	0.0095 (7)	−0.0035 (6)
S2	0.0849 (10)	0.0486 (7)	0.0511 (7)	0.0102 (6)	−0.0063 (7)	−0.0171 (6)
S3	0.0966 (11)	0.0592 (7)	0.0505 (8)	0.0205 (7)	−0.0063 (7)	−0.0174 (6)
S4	0.0921 (11)	0.0503 (7)	0.0503 (8)	0.0133 (7)	0.0025 (7)	−0.0019 (5)

### Geometric parameters (Å, º)

**Table:**

C1—C6	1.368 (6)	C47—H47	0.9300
C1—C2	1.375 (6)	C48—C49	1.384 (6)
C1—H1	0.9300	C48—H48	0.9300
C2—C3	1.367 (7)	C49—C50	1.368 (5)
C2—H2	0.9300	C49—H49	0.9300
C3—C4	1.365 (7)	C50—C51	1.497 (5)
C3—H3	0.9300	C51—N9	1.447 (4)
C4—C5	1.383 (6)	C51—H51A	0.9700
C4—H4	0.9300	C51—H51B	0.9700
C5—C6	1.374 (5)	C52—C53	1.379 (5)
C5—H5	0.9300	C52—C57	1.390 (5)
C6—C7	1.506 (5)	C52—N9	1.409 (5)
C7—N1	1.450 (5)	C53—C54	1.382 (6)
C7—H7A	0.9700	C53—H53	0.9300
C7—H7B	0.9700	C54—C55	1.372 (6)
C8—C9	1.375 (5)	C54—H54	0.9300
C8—C13	1.383 (5)	C55—C56	1.385 (5)
C8—N1	1.410 (5)	C55—H55	0.9300
C9—C10	1.368 (6)	C56—C57	1.374 (5)
C9—H9	0.9300	C56—H56	0.9300
C10—C11	1.370 (6)	C57—C58	1.443 (5)
C10—H10	0.9300	C58—N10	1.282 (4)
C11—C12	1.388 (5)	C58—C59	1.502 (5)
C11—H11	0.9300	C59—O3	1.211 (4)
C12—C13	1.372 (5)	C59—N9	1.363 (5)
C12—H12	0.9300	C60—N12	1.322 (4)
C13—C14	1.439 (5)	C60—N11	1.364 (4)
C14—N2	1.279 (4)	C60—S3	1.648 (4)
C14—C15	1.496 (5)	C61—C62	1.366 (5)
C15—O1	1.219 (5)	C61—C66	1.367 (5)
C15—N1	1.370 (5)	C61—N12	1.418 (4)
C16—N4	1.331 (4)	C62—C63	1.365 (5)
C16—N3	1.364 (4)	C62—H62	0.9300
C16—S1	1.648 (4)	C63—C64	1.354 (6)
C17—C22	1.351 (5)	C63—H63	0.9300
C17—C18	1.357 (5)	C64—C65	1.354 (6)
C17—N4	1.413 (4)	C64—H64	0.9300
C18—C19	1.393 (6)	C65—C66	1.382 (5)
C18—H18	0.9300	C65—H65	0.9300
C19—C20	1.351 (7)	C66—H66	0.9300
C19—H19	0.9300	C67—C72	1.350 (5)
C20—C21	1.348 (7)	C67—C68	1.370 (7)
C20—H20	0.9300	C67—H67	0.9300
C21—C22	1.388 (6)	C68—C69	1.337 (7)
C21—H21	0.9300	C68—H68	0.9300
C22—H22	0.9300	C69—C70	1.340 (7)
C23—C28	1.359 (6)	C69—H69	0.9300
C23—C24	1.363 (8)	C70—C71	1.393 (6)
C23—H23	0.9300	C70—H70	0.9300
C24—C25	1.348 (9)	C71—C72	1.360 (5)
C24—H24	0.9300	C71—H71	0.9300
C25—C26	1.352 (8)	C72—C73	1.498 (5)
C25—H25	0.9300	C73—N13	1.439 (4)
C26—C27	1.370 (7)	C73—H73A	0.9700
C26—H26	0.9300	C73—H73B	0.9700
C27—C28	1.363 (6)	C74—C79	1.376 (5)
C27—H27	0.9300	C74—C75	1.378 (5)
C28—C29	1.501 (5)	C74—N13	1.411 (5)
C29—N5	1.436 (5)	C75—C76	1.374 (6)
C29—H29A	0.9700	C75—H75	0.9300
C29—H29B	0.9700	C76—C77	1.374 (6)
C30—C35	1.380 (5)	C76—H76	0.9300
C30—C31	1.381 (5)	C77—C78	1.380 (5)
C30—N5	1.405 (5)	C77—H77	0.9300
C31—C32	1.369 (6)	C78—C79	1.375 (5)
C31—H31	0.9300	C78—H78	0.9300
C32—C33	1.379 (6)	C79—C80	1.446 (5)
C32—H32	0.9300	C80—N14	1.284 (4)
C33—C34	1.381 (5)	C80—C81	1.494 (5)
C33—H33	0.9300	C81—O4	1.211 (4)
C34—C35	1.371 (5)	C81—N13	1.360 (5)
C34—H34	0.9300	C82—N16	1.333 (4)
C35—C36	1.445 (5)	C82—N15	1.365 (4)
C36—N6	1.284 (4)	C82—S4	1.646 (4)
C36—C37	1.493 (5)	C83—C84	1.365 (5)
C37—O2	1.215 (4)	C83—C88	1.382 (5)
C37—N5	1.370 (5)	C83—N16	1.409 (4)
C38—N8	1.331 (4)	C84—C85	1.381 (6)
C38—N7	1.368 (4)	C84—H84	0.9300
C38—S2	1.649 (4)	C85—C86	1.363 (6)
C39—C40	1.365 (5)	C85—H85	0.9300
C39—C44	1.366 (5)	C86—C87	1.349 (6)
C39—N8	1.416 (5)	C86—H86	0.9300
C40—C41	1.368 (6)	C87—C88	1.370 (5)
C40—H40	0.9300	C87—H87	0.9300
C41—C42	1.375 (7)	C88—H88	0.9300
C41—H41	0.9300	N2—N3	1.349 (4)
C42—C43	1.346 (7)	N3—H3A	0.8600
C42—H42	0.9300	N4—H4A	0.8600
C43—C44	1.373 (6)	N6—N7	1.345 (4)
C43—H43	0.9300	N7—H7	0.8600
C44—H44	0.9300	N8—H8	0.8600
C45—C46	1.360 (6)	N10—N11	1.348 (4)
C45—C50	1.378 (6)	N11—H11A	0.8600
C45—H45	0.9300	N12—H12A	0.8600
C46—C47	1.361 (7)	N14—N15	1.352 (4)
C46—H46	0.9300	N15—H15	0.8600
C47—C48	1.361 (6)	N16—H16	0.8600
			
C6—C1—C2	121.2 (5)	C50—C51—H51A	108.6
C6—C1—H1	119.4	N9—C51—H51B	108.6
C2—C1—H1	119.4	C50—C51—H51B	108.6
C3—C2—C1	119.6 (5)	H51A—C51—H51B	107.6
C3—C2—H2	120.2	C53—C52—C57	121.6 (4)
C1—C2—H2	120.2	C53—C52—N9	128.1 (4)
C4—C3—C2	120.2 (5)	C57—C52—N9	110.2 (4)
C4—C3—H3	119.9	C52—C53—C54	116.6 (4)
C2—C3—H3	119.9	C52—C53—H53	121.7
C3—C4—C5	119.7 (5)	C54—C53—H53	121.7
C3—C4—H4	120.1	C55—C54—C53	122.4 (4)
C5—C4—H4	120.1	C55—C54—H54	118.8
C6—C5—C4	120.6 (5)	C53—C54—H54	118.8
C6—C5—H5	119.7	C54—C55—C56	120.5 (4)
C4—C5—H5	119.7	C54—C55—H55	119.8
C1—C6—C5	118.6 (4)	C56—C55—H55	119.8
C1—C6—C7	117.6 (4)	C57—C56—C55	118.1 (4)
C5—C6—C7	123.8 (4)	C57—C56—H56	120.9
N1—C7—C6	114.9 (4)	C55—C56—H56	120.9
N1—C7—H7A	108.5	C56—C57—C52	120.7 (4)
C6—C7—H7A	108.5	C56—C57—C58	132.8 (4)
N1—C7—H7B	108.5	C52—C57—C58	106.5 (4)
C6—C7—H7B	108.5	N10—C58—C57	125.1 (4)
H7A—C7—H7B	107.5	N10—C58—C59	127.9 (4)
C9—C8—C13	121.4 (5)	C57—C58—C59	107.0 (4)
C9—C8—N1	128.7 (5)	O3—C59—N9	126.7 (4)
C13—C8—N1	109.9 (4)	O3—C59—C58	127.5 (4)
C10—C9—C8	117.5 (5)	N9—C59—C58	105.9 (4)
C10—C9—H9	121.3	N12—C60—N11	114.4 (3)
C8—C9—H9	121.3	N12—C60—S3	128.4 (3)
C9—C10—C11	121.6 (5)	N11—C60—S3	117.2 (3)
C9—C10—H10	119.2	C62—C61—C66	118.6 (4)
C11—C10—H10	119.2	C62—C61—N12	117.8 (4)
C10—C11—C12	121.2 (5)	C66—C61—N12	123.6 (4)
C10—C11—H11	119.4	C63—C62—C61	121.6 (4)
C12—C11—H11	119.4	C63—C62—H62	119.2
C13—C12—C11	117.4 (4)	C61—C62—H62	119.2
C13—C12—H12	121.3	C64—C63—C62	119.6 (4)
C11—C12—H12	121.3	C64—C63—H63	120.2
C12—C13—C8	120.9 (4)	C62—C63—H63	120.2
C12—C13—C14	132.4 (4)	C63—C64—C65	120.0 (4)
C8—C13—C14	106.7 (4)	C63—C64—H64	120.0
N2—C14—C13	125.1 (4)	C65—C64—H64	120.0
N2—C14—C15	127.5 (4)	C64—C65—C66	120.6 (4)
C13—C14—C15	107.4 (4)	C64—C65—H65	119.7
O1—C15—N1	126.7 (4)	C66—C65—H65	119.7
O1—C15—C14	128.1 (4)	C61—C66—C65	119.6 (4)
N1—C15—C14	105.2 (4)	C61—C66—H66	120.2
N4—C16—N3	113.6 (3)	C65—C66—H66	120.2
N4—C16—S1	128.4 (3)	C72—C67—C68	121.5 (5)
N3—C16—S1	118.0 (3)	C72—C67—H67	119.2
C22—C17—C18	120.1 (4)	C68—C67—H67	119.2
C22—C17—N4	124.9 (4)	C69—C68—C67	120.1 (6)
C18—C17—N4	115.0 (4)	C69—C68—H68	119.9
C17—C18—C19	120.2 (5)	C67—C68—H68	119.9
C17—C18—H18	119.9	C68—C69—C70	120.2 (6)
C19—C18—H18	119.9	C68—C69—H69	119.9
C20—C19—C18	119.5 (5)	C70—C69—H69	119.9
C20—C19—H19	120.2	C69—C70—C71	119.7 (6)
C18—C19—H19	120.2	C69—C70—H70	120.1
C21—C20—C19	120.0 (5)	C71—C70—H70	120.1
C21—C20—H20	120.0	C72—C71—C70	120.4 (5)
C19—C20—H20	120.0	C72—C71—H71	119.8
C20—C21—C22	120.9 (5)	C70—C71—H71	119.8
C20—C21—H21	119.5	C67—C72—C71	117.9 (4)
C22—C21—H21	119.5	C67—C72—C73	118.8 (4)
C17—C22—C21	119.3 (5)	C71—C72—C73	123.3 (4)
C17—C22—H22	120.3	N13—C73—C72	115.2 (3)
C21—C22—H22	120.3	N13—C73—H73A	108.5
C28—C23—C24	120.4 (6)	C72—C73—H73A	108.5
C28—C23—H23	119.8	N13—C73—H73B	108.5
C24—C23—H23	119.8	C72—C73—H73B	108.5
C25—C24—C23	121.2 (7)	H73A—C73—H73B	107.5
C25—C24—H24	119.4	C79—C74—C75	121.2 (4)
C23—C24—H24	119.4	C79—C74—N13	110.0 (4)
C24—C25—C26	119.0 (7)	C75—C74—N13	128.8 (4)
C24—C25—H25	120.5	C76—C75—C74	117.0 (4)
C26—C25—H25	120.5	C76—C75—H75	121.5
C25—C26—C27	120.3 (7)	C74—C75—H75	121.5
C25—C26—H26	119.8	C77—C76—C75	122.6 (4)
C27—C26—H26	119.8	C77—C76—H76	118.7
C28—C27—C26	120.7 (6)	C75—C76—H76	118.7
C28—C27—H27	119.6	C76—C77—C78	119.8 (4)
C26—C27—H27	119.6	C76—C77—H77	120.1
C23—C28—C27	118.4 (5)	C78—C77—H77	120.1
C23—C28—C29	117.9 (5)	C79—C78—C77	118.4 (4)
C27—C28—C29	123.7 (4)	C79—C78—H78	120.8
N5—C29—C28	114.9 (4)	C77—C78—H78	120.8
N5—C29—H29A	108.6	C78—C79—C74	121.0 (4)
C28—C29—H29A	108.6	C78—C79—C80	132.2 (4)
N5—C29—H29B	108.6	C74—C79—C80	106.8 (4)
C28—C29—H29B	108.6	N14—C80—C79	125.1 (4)
H29A—C29—H29B	107.5	N14—C80—C81	128.1 (4)
C35—C30—C31	121.2 (5)	C79—C80—C81	106.8 (4)
C35—C30—N5	110.5 (4)	O4—C81—N13	126.2 (4)
C31—C30—N5	128.3 (5)	O4—C81—C80	128.0 (4)
C32—C31—C30	117.2 (5)	N13—C81—C80	105.8 (4)
C32—C31—H31	121.4	N16—C82—N15	113.8 (3)
C30—C31—H31	121.4	N16—C82—S4	128.9 (3)
C31—C32—C33	121.8 (5)	N15—C82—S4	117.3 (3)
C31—C32—H32	119.1	C84—C83—C88	119.0 (4)
C33—C32—H32	119.1	C84—C83—N16	117.2 (4)
C32—C33—C34	120.8 (5)	C88—C83—N16	123.8 (4)
C32—C33—H33	119.6	C83—C84—C85	120.4 (4)
C34—C33—H33	119.6	C83—C84—H84	119.8
C35—C34—C33	117.6 (5)	C85—C84—H84	119.8
C35—C34—H34	121.2	C86—C85—C84	119.9 (4)
C33—C34—H34	121.2	C86—C85—H85	120.1
C34—C35—C30	121.3 (4)	C84—C85—H85	120.1
C34—C35—C36	132.4 (4)	C87—C86—C85	120.0 (4)
C30—C35—C36	106.3 (4)	C87—C86—H86	120.0
N6—C36—C35	124.6 (4)	C85—C86—H86	120.0
N6—C36—C37	128.0 (4)	C86—C87—C88	120.9 (4)
C35—C36—C37	107.4 (4)	C86—C87—H87	119.6
O2—C37—N5	126.7 (4)	C88—C87—H87	119.6
O2—C37—C36	127.9 (4)	C87—C88—C83	119.9 (4)
N5—C37—C36	105.4 (4)	C87—C88—H88	120.1
N8—C38—N7	113.0 (3)	C83—C88—H88	120.1
N8—C38—S2	128.7 (3)	C15—N1—C8	110.7 (4)
N7—C38—S2	118.3 (3)	C15—N1—C7	123.4 (4)
C40—C39—C44	119.9 (4)	C8—N1—C7	125.7 (4)
C40—C39—N8	115.7 (4)	C14—N2—N3	117.9 (3)
C44—C39—N8	124.4 (4)	N2—N3—C16	120.0 (3)
C39—C40—C41	120.2 (5)	N2—N3—H3A	120.0
C39—C40—H40	119.9	C16—N3—H3A	120.0
C41—C40—H40	119.9	C16—N4—C17	130.9 (3)
C40—C41—C42	120.1 (5)	C16—N4—H4A	114.5
C40—C41—H41	120.0	C17—N4—H4A	114.5
C42—C41—H41	120.0	C37—N5—C30	110.4 (4)
C43—C42—C41	119.0 (5)	C37—N5—C29	123.7 (4)
C43—C42—H42	120.5	C30—N5—C29	125.7 (4)
C41—C42—H42	120.5	C36—N6—N7	117.0 (3)
C42—C43—C44	121.7 (5)	N6—N7—C38	120.3 (3)
C42—C43—H43	119.2	N6—N7—H7	119.8
C44—C43—H43	119.2	C38—N7—H7	119.8
C39—C44—C43	119.1 (5)	C38—N8—C39	131.3 (3)
C39—C44—H44	120.5	C38—N8—H8	114.3
C43—C44—H44	120.5	C39—N8—H8	114.3
C46—C45—C50	121.6 (5)	C59—N9—C52	110.4 (3)
C46—C45—H45	119.2	C59—N9—C51	123.4 (4)
C50—C45—H45	119.2	C52—N9—C51	125.8 (4)
C45—C46—C47	120.2 (5)	C58—N10—N11	117.3 (3)
C45—C46—H46	119.9	N10—N11—C60	120.9 (3)
C47—C46—H46	119.9	N10—N11—H11A	119.5
C48—C47—C46	119.5 (5)	C60—N11—H11A	119.5
C48—C47—H47	120.3	C60—N12—C61	130.0 (3)
C46—C47—H47	120.3	C60—N12—H12A	115.0
C47—C48—C49	120.4 (5)	C61—N12—H12A	115.0
C47—C48—H48	119.8	C81—N13—C74	110.6 (3)
C49—C48—H48	119.8	C81—N13—C73	124.2 (4)
C50—C49—C48	120.5 (5)	C74—N13—C73	124.9 (4)
C50—C49—H49	119.7	C80—N14—N15	117.1 (3)
C48—C49—H49	119.7	N14—N15—C82	121.0 (3)
C49—C50—C45	117.8 (4)	N14—N15—H15	119.5
C49—C50—C51	124.1 (4)	C82—N15—H15	119.5
C45—C50—C51	118.0 (4)	C82—N16—C83	130.0 (3)
N9—C51—C50	114.5 (3)	C82—N16—H16	115.0
N9—C51—H51A	108.6	C83—N16—H16	115.0
			
C6—C1—C2—C3	−0.7 (8)	C67—C68—C69—C70	−2.3 (10)
C1—C2—C3—C4	0.3 (9)	C68—C69—C70—C71	0.3 (9)
C2—C3—C4—C5	0.2 (9)	C69—C70—C71—C72	2.1 (8)
C3—C4—C5—C6	−0.3 (8)	C68—C67—C72—C71	0.4 (7)
C2—C1—C6—C5	0.6 (7)	C68—C67—C72—C73	−180.0 (5)
C2—C1—C6—C7	−179.4 (4)	C70—C71—C72—C67	−2.4 (7)
C4—C5—C6—C1	−0.1 (7)	C70—C71—C72—C73	178.0 (4)
C4—C5—C6—C7	179.9 (4)	C67—C72—C73—N13	173.9 (4)
C1—C6—C7—N1	154.4 (4)	C71—C72—C73—N13	−6.5 (6)
C5—C6—C7—N1	−25.6 (6)	C79—C74—C75—C76	1.4 (6)
C13—C8—C9—C10	1.3 (6)	N13—C74—C75—C76	−178.8 (4)
N1—C8—C9—C10	−178.8 (4)	C74—C75—C76—C77	−0.6 (7)
C8—C9—C10—C11	−0.6 (7)	C75—C76—C77—C78	−0.5 (7)
C9—C10—C11—C12	0.0 (7)	C76—C77—C78—C79	0.8 (6)
C10—C11—C12—C13	−0.2 (6)	C77—C78—C79—C74	0.0 (6)
C11—C12—C13—C8	0.9 (6)	C77—C78—C79—C80	−179.0 (4)
C11—C12—C13—C14	−179.0 (4)	C75—C74—C79—C78	−1.2 (6)
C9—C8—C13—C12	−1.5 (6)	N13—C74—C79—C78	179.0 (4)
N1—C8—C13—C12	178.6 (4)	C75—C74—C79—C80	178.1 (4)
C9—C8—C13—C14	178.4 (4)	N13—C74—C79—C80	−1.7 (4)
N1—C8—C13—C14	−1.4 (5)	C78—C79—C80—N14	2.4 (7)
C12—C13—C14—N2	2.6 (7)	C74—C79—C80—N14	−176.7 (4)
C8—C13—C14—N2	−177.3 (4)	C78—C79—C80—C81	−179.7 (4)
C12—C13—C14—C15	−179.2 (4)	C74—C79—C80—C81	1.2 (4)
C8—C13—C14—C15	0.9 (4)	N14—C80—C81—O4	−2.9 (7)
N2—C14—C15—O1	−1.1 (7)	C79—C80—C81—O4	179.3 (4)
C13—C14—C15—O1	−179.2 (4)	N14—C80—C81—N13	177.6 (4)
N2—C14—C15—N1	178.1 (4)	C79—C80—C81—N13	−0.2 (4)
C13—C14—C15—N1	−0.1 (4)	C88—C83—C84—C85	0.5 (7)
C22—C17—C18—C19	1.3 (7)	N16—C83—C84—C85	178.8 (4)
N4—C17—C18—C19	−178.8 (4)	C83—C84—C85—C86	−0.8 (8)
C17—C18—C19—C20	−0.9 (8)	C84—C85—C86—C87	1.1 (9)
C18—C19—C20—C21	0.1 (9)	C85—C86—C87—C88	−1.2 (8)
C19—C20—C21—C22	0.2 (9)	C86—C87—C88—C83	1.0 (8)
C18—C17—C22—C21	−0.9 (7)	C84—C83—C88—C87	−0.6 (7)
N4—C17—C22—C21	179.1 (4)	N16—C83—C88—C87	−178.7 (4)
C20—C21—C22—C17	0.2 (8)	O1—C15—N1—C8	178.4 (4)
C28—C23—C24—C25	−1.4 (10)	C14—C15—N1—C8	−0.8 (4)
C23—C24—C25—C26	1.7 (12)	O1—C15—N1—C7	3.6 (7)
C24—C25—C26—C27	0.0 (11)	C14—C15—N1—C7	−175.5 (3)
C25—C26—C27—C28	−2.0 (9)	C9—C8—N1—C15	−178.4 (4)
C24—C23—C28—C27	−0.6 (8)	C13—C8—N1—C15	1.5 (5)
C24—C23—C28—C29	179.8 (5)	C9—C8—N1—C7	−3.8 (7)
C26—C27—C28—C23	2.3 (7)	C13—C8—N1—C7	176.0 (4)
C26—C27—C28—C29	−178.2 (5)	C6—C7—N1—C15	−98.7 (5)
C23—C28—C29—N5	−180.0 (4)	C6—C7—N1—C8	87.4 (5)
C27—C28—C29—N5	0.5 (6)	C13—C14—N2—N3	177.7 (3)
C35—C30—C31—C32	−2.0 (7)	C15—C14—N2—N3	−0.1 (6)
N5—C30—C31—C32	177.9 (4)	C14—N2—N3—C16	179.3 (4)
C30—C31—C32—C33	1.5 (7)	N4—C16—N3—N2	−5.8 (5)
C31—C32—C33—C34	−0.1 (7)	S1—C16—N3—N2	174.6 (3)
C32—C33—C34—C35	−0.9 (7)	N3—C16—N4—C17	174.5 (4)
C33—C34—C35—C30	0.4 (6)	S1—C16—N4—C17	−6.0 (7)
C33—C34—C35—C36	179.7 (4)	C22—C17—N4—C16	23.1 (7)
C31—C30—C35—C34	1.1 (6)	C18—C17—N4—C16	−156.9 (4)
N5—C30—C35—C34	−178.8 (4)	O2—C37—N5—C30	−179.7 (4)
C31—C30—C35—C36	−178.3 (4)	C36—C37—N5—C30	1.1 (4)
N5—C30—C35—C36	1.7 (5)	O2—C37—N5—C29	−3.6 (7)
C34—C35—C36—N6	−2.1 (7)	C36—C37—N5—C29	177.1 (3)
C30—C35—C36—N6	177.3 (4)	C35—C30—N5—C37	−1.8 (5)
C34—C35—C36—C37	179.6 (5)	C31—C30—N5—C37	178.2 (4)
C30—C35—C36—C37	−1.0 (4)	C35—C30—N5—C29	−177.8 (4)
N6—C36—C37—O2	2.5 (7)	C31—C30—N5—C29	2.3 (7)
C35—C36—C37—O2	−179.3 (4)	C28—C29—N5—C37	105.0 (5)
N6—C36—C37—N5	−178.3 (4)	C28—C29—N5—C30	−79.5 (5)
C35—C36—C37—N5	0.0 (4)	C35—C36—N6—N7	−177.8 (4)
C44—C39—C40—C41	0.9 (7)	C37—C36—N6—N7	0.2 (6)
N8—C39—C40—C41	−179.8 (4)	C36—N6—N7—C38	−178.5 (3)
C39—C40—C41—C42	−0.8 (8)	N8—C38—N7—N6	5.5 (5)
C40—C41—C42—C43	1.0 (8)	S2—C38—N7—N6	−174.5 (3)
C41—C42—C43—C44	−1.3 (8)	N7—C38—N8—C39	−171.3 (4)
C40—C39—C44—C43	−1.2 (7)	S2—C38—N8—C39	8.8 (7)
N8—C39—C44—C43	179.6 (4)	C40—C39—N8—C38	155.9 (4)
C42—C43—C44—C39	1.4 (8)	C44—C39—N8—C38	−24.8 (7)
C50—C45—C46—C47	1.8 (8)	O3—C59—N9—C52	−178.1 (4)
C45—C46—C47—C48	−1.6 (9)	C58—C59—N9—C52	1.4 (4)
C46—C47—C48—C49	0.5 (8)	O3—C59—N9—C51	−5.1 (7)
C47—C48—C49—C50	0.5 (7)	C58—C59—N9—C51	174.4 (3)
C48—C49—C50—C45	−0.4 (6)	C53—C52—N9—C59	178.5 (4)
C48—C49—C50—C51	−180.0 (4)	C57—C52—N9—C59	−2.4 (4)
C46—C45—C50—C49	−0.8 (7)	C53—C52—N9—C51	5.6 (6)
C46—C45—C50—C51	178.9 (4)	C57—C52—N9—C51	−175.2 (3)
C49—C50—C51—N9	26.9 (6)	C50—C51—N9—C59	98.9 (4)
C45—C50—C51—N9	−152.7 (4)	C50—C51—N9—C52	−89.1 (5)
C57—C52—C53—C54	−0.4 (6)	C57—C58—N10—N11	−177.5 (3)
N9—C52—C53—C54	178.7 (4)	C59—C58—N10—N11	0.2 (6)
C52—C53—C54—C55	0.0 (6)	C58—N10—N11—C60	−179.5 (3)
C53—C54—C55—C56	0.6 (7)	N12—C60—N11—N10	5.6 (5)
C54—C55—C56—C57	−0.9 (6)	S3—C60—N11—N10	−175.9 (3)
C55—C56—C57—C52	0.5 (6)	N11—C60—N12—C61	−177.6 (4)
C55—C56—C57—C58	178.7 (4)	S3—C60—N12—C61	4.1 (6)
C53—C52—C57—C56	0.1 (6)	C62—C61—N12—C60	−153.5 (4)
N9—C52—C57—C56	−179.1 (4)	C66—C61—N12—C60	29.0 (7)
C53—C52—C57—C58	−178.5 (4)	O4—C81—N13—C74	179.7 (4)
N9—C52—C57—C58	2.3 (4)	C80—C81—N13—C74	−0.8 (4)
C56—C57—C58—N10	−1.6 (7)	O4—C81—N13—C73	5.9 (7)
C52—C57—C58—N10	176.7 (4)	C80—C81—N13—C73	−174.6 (3)
C56—C57—C58—C59	−179.7 (4)	C79—C74—N13—C81	1.7 (5)
C52—C57—C58—C59	−1.4 (4)	C75—C74—N13—C81	−178.1 (4)
N10—C58—C59—O3	1.5 (7)	C79—C74—N13—C73	175.4 (4)
C57—C58—C59—O3	179.5 (4)	C75—C74—N13—C73	−4.4 (7)
N10—C58—C59—N9	−178.0 (4)	C72—C73—N13—C81	−104.6 (5)
C57—C58—C59—N9	0.0 (4)	C72—C73—N13—C74	82.5 (5)
C66—C61—C62—C63	−1.5 (7)	C79—C80—N14—N15	177.8 (3)
N12—C61—C62—C63	−179.2 (4)	C81—C80—N14—N15	0.4 (6)
C61—C62—C63—C64	1.4 (8)	C80—N14—N15—C82	178.7 (4)
C62—C63—C64—C65	−1.1 (8)	N16—C82—N15—N14	−5.7 (5)
C63—C64—C65—C66	1.1 (8)	S4—C82—N15—N14	175.7 (3)
C62—C61—C66—C65	1.5 (7)	N15—C82—N16—C83	176.4 (4)
N12—C61—C66—C65	179.0 (4)	S4—C82—N16—C83	−5.2 (7)
C64—C65—C66—C61	−1.3 (7)	C84—C83—N16—C82	154.5 (4)
C72—C67—C68—C69	2.0 (9)	C88—C83—N16—C82	−27.3 (7)

### Hydrogen-bond geometry (Å, º)

Cg2 and Cg12 are the centroids of rings C1–C6 (molecule *A*) and C45–C50 (molecule *C*), respectively.

**Table:**

*D*—H···*A*	*D*—H	H···*A*	*D*···*A*	*D*—H···*A*
N3—H3*A*···O1	0.86	2.11	2.777 (4)	134
N7—H7···O2	0.86	2.10	2.773 (4)	135
N11—H11*A*···O3	0.86	2.09	2.771 (4)	135
N15—H15···O4	0.86	2.10	2.779 (4)	135
N4—H4*A*···N2	0.86	2.13	2.575 (4)	112
N8—H8···N6	0.86	2.12	2.570 (5)	112
N12—H12*A*···N10	0.86	2.17	2.606 (4)	111
N16—H16···N14	0.86	2.16	2.603 (4)	111
C22—H22···S1	0.93	2.58	3.203 (5)	125
C44—H44···S2	0.93	2.61	3.224 (5)	124
C66—H66···S3	0.93	2.67	3.217 (4)	119
C88—H88···S4	0.93	2.67	3.227 (4)	120
C10—H10···S3^i^	0.93	2.80	3.697 (5)	164
C21—H21···S4^ii^	0.93	2.80	3.710 (5)	166
C32—H32···S1^iii^	0.93	2.74	3.648 (6)	166
C43—H43···S3^iv^	0.93	2.81	3.712 (6)	165
C76—H76···S2^i^	0.93	2.84	3.721 (5)	159
C41—H41···*Cg*2^v^	0.93	2.99	3.788 (6)	145
C78—H78···*Cg*12^i^	0.93	2.95	3.717 (5)	141

Symmetry codes: (i) *x*−1, *y*+1, *z*; (ii) −*x*+1, −*y*, −*z*+2; (iii) *x*, *y*+1, *z*; (iv) −*x*+2, −*y*, −*z*+1; (v) −*x*+1, −*y*+1, −*z*+1.
