# Supplementary figures and images for: Crystal structure of (Z)-2-(1-benzyl-2-oxoindolin-3-yl­idene)-N-phenyl­hydra­zine-1-carbo­thio­amide
Source: Acta Crystallogr E Crystallogr Commun. 2015 Feb 7;71(Pt 3):o160–1. doi: 10.1107/S2056989015002248 (PMC4350691; doi:10.1107/S2056989015002248)

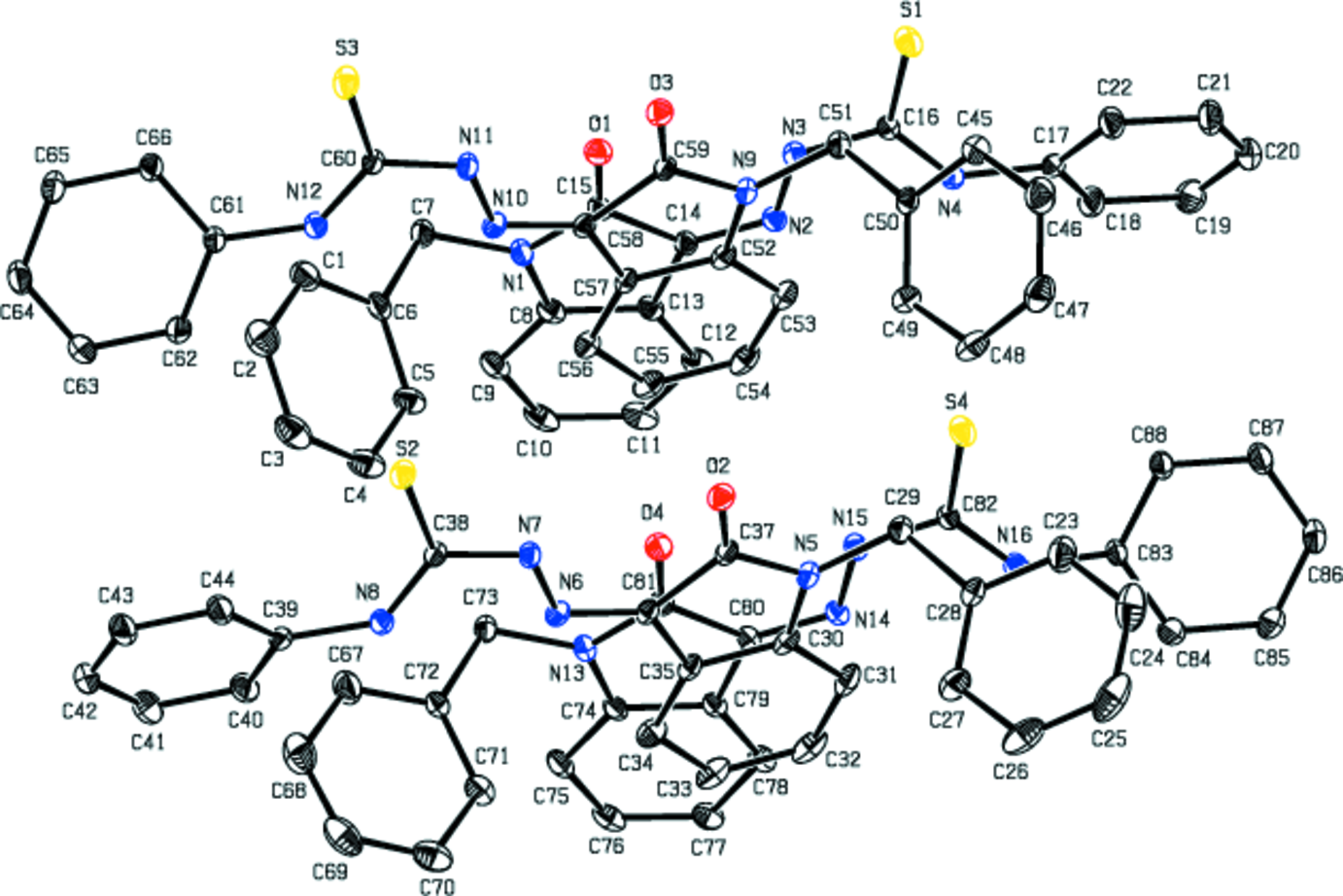

Supplement: Supplementary file 4 [file e-71-0o160-fig1.tif]

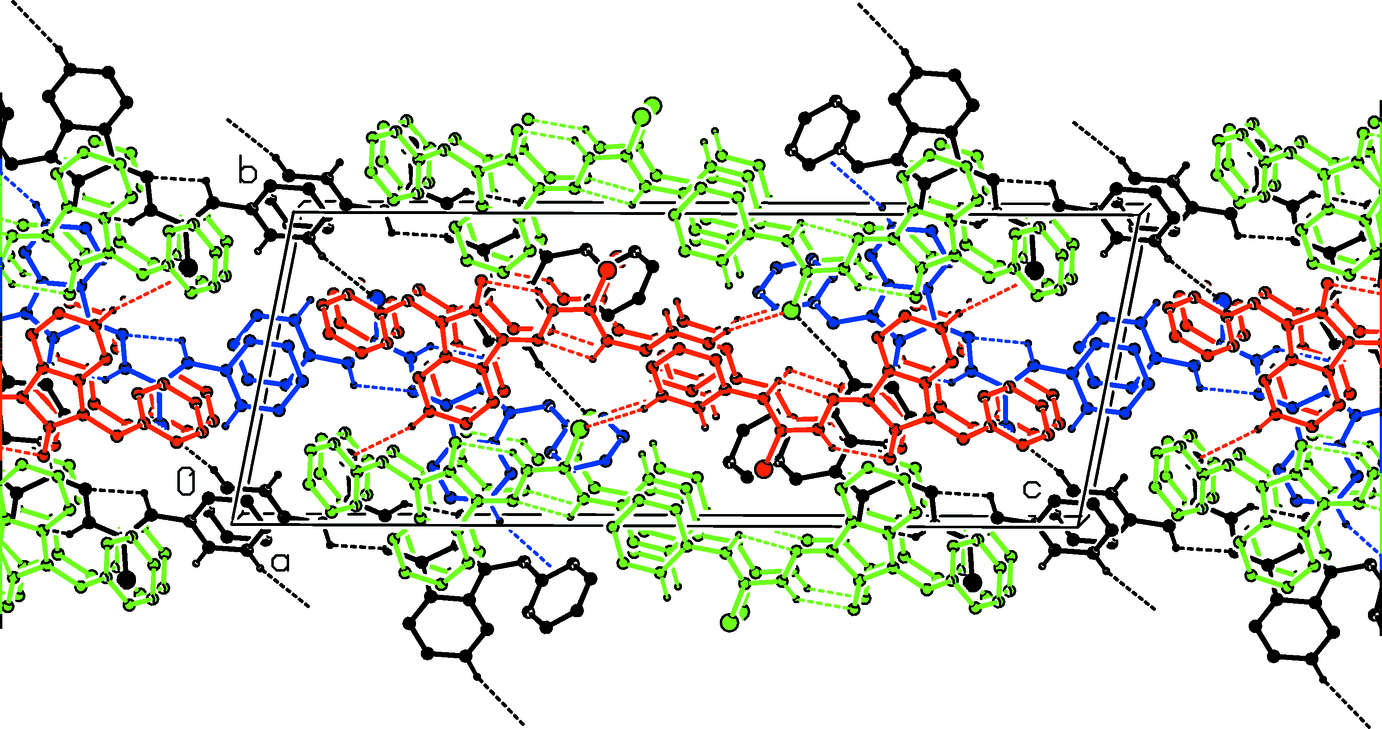

Supplement: Supplementary file 5 [file e-71-0o160-fig2.tif]
